# Reasons for Low Pandemic H1N1 2009 Vaccine Acceptance within a College Sample

**DOI:** 10.1155/2012/242518

**Published:** 2012-11-28

**Authors:** Russell D. Ravert, Linda Y. Fu, Gregory D. Zimet

**Affiliations:** ^1^Department of Human Development & Family Studies, University of Missouri, Columbia, MO 65211, USA; ^2^Goldberg Center for Community Pediatric Health, Children's National Medical Center, Washington, DC 20010, USA; ^3^Section of Adolescent Medicine, Indiana University School of Medicine, Indianapolis, IN 46202, USA

## Abstract

This study examined health beliefs associated with novel influenza A (H1N1) immunization among US college undergraduates during the 2009-2010 pandemic. Undergraduates (ages 18–24 years) from a large Midwestern University were invited to complete an online survey during March, 2010, five months after H1N1 vaccines became available. Survey items measured H1N1 vaccine history and H1N1-related attitudes based on the health belief literature. Logistic regression was used to identify attitudes associated with having received an H1N1 vaccine, and thematic analysis of student comments was conducted to further understand influences on vaccine decisions. Among the 296 students who participated in the survey, 15.2% reported having received an H1N1 vaccine. In regression analysis, H1N1 immunization was associated with seasonal flu vaccine history, perceived vaccine effectiveness, perceived obstacles to vaccination, and vaccine safety concerns. Qualitative results illustrate the relationship of beliefs to vaccine decisions, particularly in demonstrating that students often held concerns that vaccine could cause H1N1 or side effects. Vaccine safety, efficacy, and obstacles to immunization were major considerations in deciding whether to accept the H1N1 pandemic vaccine. Therefore, focusing on those aspects might be especially useful in future vaccine efforts within the college population.

## 1. Introduction 

College students are at especially high risk of contracting and passing on infectious diseases, due in part to dormitory living [1] and large social gatherings [2], and influenza is a known health and academic concern for that population [3]. When novel influenza A (H1N1) pandemic vaccines were made available in 2009, young people up to 25 years of age were targeted for vaccination due to their high rate of H1N1 infection and complications [4]. For these reasons, the Centers for Disease Control and Prevention [5] and the American College Health Association [6] issued guidelines for colleges regarding the evolving H1N1 flu pandemic, recommending strategies for containing the epidemic on campuses including facilitating student vaccinations. 

However, by Fall, 2009, anecdotal reports indicated that students were choosing to forgo H1N1 vaccination even after concentrated campus vaccine promotion efforts [7]. Although no definitive data regarding college H1N1 vaccine acceptance rates are available, several published studies utilizing college samples suggest that protective behaviors, intentions to be vaccinated and actual H1N1 vaccine uptake among US and international students remained relatively low throughout the pandemic. In surveys conducted at an Australian college between June and September of 2009, 76.8% of students reported not having made any changes to the way they lived due to H1N1 [8]. A Fall, 2009, survey conducted at a US University found that only 15.8% of students intended to be vaccinated [9]. Similarly, in surveys of college students in Italy, Israel, and Turkey, less than 25%, 13.9%, and 7.2%, respectively, reported willingness to be vaccinated against H1N1 [10–12]. 

Reports of actual college H1N1 vaccination rates ranged from 8% among Greek medical students [13] to 12.3% of students in a US college [14] and 12.7% of students at an Indian university [15] By the end of April 2010, only 8% of students at colleges and universities participating in the American College Health Association's Pandemic Influenza Surveillance program had reportedly been vaccinated [16]. 

The Health Belief Model [17] provides a framework that might help explain the low levels of H1N1 vaccination acceptance among college students. The model explains a given protective behavior as resulting from the degree to which an individual (a) feels susceptible to a condition, (b) perceives that condition as severe, (c) perceives benefits to taking the protective action, and (d) perceives few barriers to taking that action [18]. These four dimensions of health beliefs have been demonstrated to predict hypothetical and actual vaccine acceptance in previous studies [19, 20], although their relative contribution as predictors has varied across vaccine types and samples. 

In addition to weighing perceived risks and benefits, individuals often consider the attitudes of other people when deciding whether to engage in a health protective behavior (a key element of the Theory of Reasoned Action or TRA) [21]. Therefore, for example, students who believe that getting an H1N1 vaccine is valued by parents or friends may be more likely to do become vaccinated, even controlling for their own personal health beliefs. 

Finally, choices regarding protective behaviors (such as being immunized) could potentially be influenced by perceived efficacy. The Extended Parallel Process Model [22] asserts that assessing a threat as dangerous results in adaptive preventative behavior only when accompanied by a belief that taking action will effectively avoid the danger. 

Prior research has demonstrated the association between college students' health beliefs and their H1N1 immunization intentions. In a sample of students attending college in Israel during the H1N1 pandemic, Teitler-Regev and colleagues [11] found intent to accept H1N1 vaccination to be associated with, (a) previous seasonal flu shot experience, (b) high perceived susceptibility, (c) high perceived severity, and (d) low perceived barriers (conceptualized as a high degree of risk associated with the H1N1 vaccine). In that study, the most common reasons for getting vaccinated was to avoid contracting H1N1, whereas the most common reasons for rejecting the vaccine was lack of knowledge regarding the vaccine's safety and lack of perceived effectiveness. The goal of the current study was, similarly, to assess the association between students' health beliefs and H1N1 vaccine decisions, but to utilize actual vaccine acceptance (rather than intention) as the outcome. 

Better understanding the relatively low vaccination rates found among college students during the 2009-2010 pandemic involves identifying specific beliefs that were associated with declining or accepting H1N1 vaccination. This information could be vital to clinicians and other health professionals hoping to design successful immunization programs when future pandemics arise. The present study used a mixed methods approach in order to identify which attitudes and beliefs were associated with college student's H1N1 vaccination decisions during the 2009-2010 pandemic. 

## 2. Materials and Methods 

Using a concurrent mixed methods research design [23], quantitative data and qualitative data were collected via the same online survey. Logistic regression analysis and thematic content analysis were conducted, with the aim to reach a degree of convergence between those quantitative and qualitative findings in order to allow a more comprehensive perspective than either method could produce independently. 

### 2.1. Data Collection

A convenience sample of 296 students (ages from 18 to 24) was recruited from four large undergraduate courses taught at a large Midwestern public university. The study received university IRB approval, and the requirement for written informed consent was waived. Instructors of four survey courses taught within a College of Human Environmental Sciences provided permission for the researcher to make a brief announcement regarding the study. Following the announcement, all students on the course rosters were sent an e-mail describing the study. Clicking on a link within the e-mail message acknowledged consent and led participants to an online survey consisting of forced-choice items and one open-ended question. To aid survey response rates, five survey participants were randomly selected to receive a $50 gift card to the University Bookstore. In total, 1,171 students were sent an invitation to participate. A reminder e-mail was sent one week before closing the survey. All surveys were completed during March, 2010, five months after H1N1 vaccines were made available to the public. 

### 2.2. Quantitative Measures

#### 2.2.1. Seasonal Influenza Vaccine History

Students responded to an item worded, “Seasonal flu vaccines are offered every year. How often do you get the annual seasonal flu vaccine?” Response options were never, seldom, sometimes, almost every year, and every year. 

#### 2.2.2. Beliefs and Attitudes

Based on a review of prior studies and measures of HBM and TRA constructs [24–27], a 21-item measure (available from the first author) was created for use in the study. The measure included 3-item scales for each of seven constructs, with high scores representing, respectively, high degrees of (a) perceived H1N1 disease susceptibility, (b) perceived H1N1 disease severity, (c) perceived efficacy of the H1N1 vaccine, (d) perceived obstacles to getting an H1N1 vaccine, (e) concern regarding the safety of H1N1 vaccination, (f) general medical dislike, and (g) H1N1 vaccination endorsement from family and friends. Response options for the 21 items were on a 5-point Likert scale from “strongly disagree” to “strongly agree.” Exploratory factor analysis of the 21 items supported a seven-factor solution with all items loading onto their respective scales as conceptualized. Reliability (Chronbach's alpha) for scales ranged from .72 to .87, and all scales approximated normal distributions. 

### 2.3. Quantitative Analysis

Listwise deletion was used in the regression analysis, resulting in 5 missing cases (1.7%), and considered acceptable. All analyses were conducted using PASW statistical software, version 18.0. 

A series of univariate logistic regressions were conducted in order to determine which individual predictors of H1N1 vaccination should be included in a multivariable logistic regression model. Predictor variables considered were sex, age, seasonal influenza vaccination history, perceived H1N1 disease susceptibility, perceived H1N1 disease severity, perceived H1N1 vaccine efficacy, perceived H1N1 vaccination obstacles, H1N1 vaccine safety concerns, general medical dislike, and H1N1 vaccination endorsement by family and friends. All predictors with a significance level of *P* < .10 in univariate logistic regression were considered for entry into an adjusted, multivariable model with H1N1 vaccination as the outcome. 

### 2.4. Qualitative Analysis

Qualitative data were collected on a single survey item that asked respondents to list any additional comments about the H1N1 vaccine or the survey. In analysis, thematic coding was used to generate a set of categories that best reflected the reasons cited by respondents for receiving or failing to receive an H1N1 vaccine. The analysis process involved three steps: (a) identifying all comments that attempted to explain the rationale behind a respondent's H1N1 vaccination decision, (b) establishing a set of thematic categories that captured ideas in those comments, and (c) coding all comments into one of the thematic categories. Comments unrelated to H1N1 decisions were not included in analysis. 

## 3. Results and Discussion 

### 3.1. Descriptive

A total of 296 respondents who met the study criteria completed the survey, representing 25.3% of students to whom an invitation was sent. All participants who began the survey completed it, with a median completion time of eight minutes. The sample averaged 19.7 years of age, with between 12% and 38% of each grade level, freshman through senior, represented. The ethnic distribution within the sample (87.5% white, 5.4% black, 3% Asian) was comparable to that found in the college in which data collection took place. However, a higher proportion of females was present in the sample (87.1%) compared with the college (71.5%). 

Forty-five students (15.2%) reported having received the H1N1 vaccine. Vaccination rates did not differ significantly by sex (16.1% of females versus 10.5% of males), *χ* ^2^ = .800, df = 1, and *P* = .371. There was no significant difference in vaccination rates between the students who self-identified as white compared with other respondents (16.0% of white versus 10.8% nonwhite), *χ* ^2^ = .660, df = 1, and *P* = .417.

### 3.2. Regression Results

In univariate regression analysis, variables meeting the *P* < .10 significance criteria and included in the multivariable model were prior seasonal influenza vaccination acceptance, perceived H1N1 disease susceptibility, perceived H1N1 disease severity, perceived H1N1 vaccine efficacy, perceived obstacles to vaccination, H1N1 vaccine safety concerns, and H1N1 vaccination endorsement by family and friends. In multivariable logistic regression after adjustment to include only variables meeting the established significance criteria, factors independently associated with H1N1 vaccine acceptance were (a) prior seasonal influenza vaccination acceptance, (b) perceived high H1N1 vaccine efficacy, (c) few perceived obstacles to obtaining an H1N1 vaccine, and (d) few H1N1 vaccine safety concerns (Table 1). Perceived H1N1 disease susceptibility, severity, and vaccine endorsement by family and friends were not significant predictors of H1N1 vaccination in the multivariable logistic regression analysis. 

### 3.3. Qualitative Results

Approximately one-third of survey participants (*n* = 98, 33.1%) chose to write a reply to the open-ended survey item, resulting in 112 comments providing reasons to receive or decline an H1N1 vaccine. Almost without exception, comments focused on reasons to refuse H1N1 vaccination (rather than reasons to accept). Only three comments expressed full endorsement of the vaccine (e.g., “I think the vaccine is a good thing for people to get”). 

Table 2 presents thematic categories and representative comments. The most common comment type expressed concern over H1N1 vaccine safety. Specifically, students cited fear of contracting H1N1 disease from the vaccine, experiencing side effects from the vaccine, or that immunization can cause viral mutation into a more virulent strain (e.g., “in the back of my mind was the theory that antibiotics and things we do to get rid of or kill viruses/pathogens cause them to adapt and become more resistant and make themselves immune to our cures”). Respondents cited a variety of sources for their safety concerns, including the experience of someone they knew (e.g., “I know of many people actually getting H1N1 from the nasal mist”), and the Internet (e.g., “There are also videos on YouTube of things that go wrong to people when they get the vaccine”).

In all six of the comments coded as* history*, respondents reported to have seldom (or never) received seasonal influenza vaccinations and considered this as an influence on the decision to refuse H1N1 vaccine (e.g., “I have never gotten a flu shot so it would be pointless for me to start now”). In comments coded in the* susceptibility* category, respondents expressed reasons for feeling unlikely to contract the H1N1 virus, including that she or he was especially healthy (e.g., “I rarely get sick and I do not feel it is necessary…”) and that H1N1 disease could be avoided though proper hygiene (e.g., “The chances of me getting the H1N1 flu aren't all that high as long as I take care of myself and sanitize.”). Comments coded as* severity* frequently compared disease from H1N1 to seasonal influenza and minimized the potential severity of H1N1 disease, (e.g., “I honestly think that H1N1 is not any different from the flu despite what the media or others might hype it up to be. I just think they're sending people into an unnecessary panic.”). Comments in the* efficacy* category included opinions that the H1N1 vaccine had not been adequately tested, that it was made available too late in the pandemic to be useful, and that vaccines in general are “just a guess” or “not fail proof.” Comments labeled* obstacles* mentioned mode of administration (injection and nasal mist), cost, and ease in obtaining the vaccine as factors impacting their H1N1 vaccination decision (e.g., “I think more people would get it on campus for free”). Within the* medical dislike* category, the most common comments involved avoidance of injections (e.g., “I'm not too fond of needles and injections”). In each of the five comments coded as* endorsement*, respondents cited physician advice not to get the vaccine (e.g., “When consulting my physician about the vaccine she did not recommend getting the live vaccination and was not planning to get it herself”). 

One additional category not included in the original scheme emerged during analysis was termed* information*. Eleven respondents claimed that lack adequate information impacted their decision about H1N1 vaccination. One student wrote, “The public received precious little fact-based information and too much of the pro- and anti-vaccination sides saying “get it” or “no do not get it”. In that kind of environment it's easiest just to maintain the status quo.” The idea that lack of knowledge and uncertainty were reasons to declining vaccination was a consistent theme in the* information* category. 

Comments offered by students who had received an H1N1 vaccine demonstrate the particular importance of perceived obstacles, vaccine safety and efficacy as influences weighing on vaccine decisions. Comments among these students included opinions that the nasal mist “was much easier than the shot,” and that the vaccine was “quick and easy.” One student noted that the vaccine “took less than 5 minutes,” and another had received a free vaccine but “won't ever pay to get the H1N1 vaccine.” Several students who received the vaccine still expressed concerns regarding the safety or efficacy of the vaccine, including the comments “I know many people actually getting H1N1 from the nasal mist” and “I got the H1N1 vaccine and became ill after getting it.” One student expressed that “most of the H1N1 virus had already passed through … by the time the vaccine arrived.” Two students reported being vaccinated because it was required in their workplace, and one student indicated that she had accepted a vaccine because she was diabetic, although she did not consider it effective. 

## 4. Conclusions 

Among our sample of 296 US undergraduates, only 15.2% reported having received an H1N1 vaccine. This finding is consistent with the relatively low acceptance rates reported in other studies of U.S. and international college samples [13, 14, 16, 28]. Our multivariable logistic regression and qualitative analyses suggest that the low rate of H1N1 vaccination within our college sample was best explained by a combination of prior vaccine experience, safety concerns regarding the vaccine, perceptions of low H1N1 vaccine efficacy, and perceived obstacles to vaccination. The outcome that prior seasonal influenza vaccination acceptance predicted H1N1 vaccine acceptance is consistent with results from previous studies [29]. However, prior acceptance of the seasonal influenza vaccine is not a guarantee of H1N1 vaccine acceptance, as demonstrated by our finding that of 36 students who reported getting the annual seasonal flu vaccine every year, 20 (55.6%) had not received an H1N1 vaccine. Recognizing the relationship between various health beliefs and H1N1 vaccine acceptance is important to develop an understanding of how future pandemic vaccines might be received. 

Unlike previous studies of college students' acceptance of vaccines* other than H1N1* [19, 20, 27, 30], we did not find perceived susceptibility to be an independent predictor of vaccine acceptance when considering additional health beliefs. Our findings are also in contrast to Teitler-Regev and colleagues [11] who found susceptibility to predict college student intentions to accept H1N1 vaccination. Rather, our findings suggest that variables other than susceptibility were primary considerations in H1N1 vaccination among our college student sample. Furthermore, while 83.8% of our respondents agreed that “college students have a high likelihood of getting infected with the H1N1 virus,” only 37.2% agreed that, “people like me are likely to get sick with H1N1 influenza.” These findings suggest that optimism bias [31] regarding avoiding the pandemic may have been a factor in some of our respondents' vaccination perceptions and decisions. 

The beliefs that best explained H1N1 vaccination acceptance were related to vaccine efficacy, perceived obstacles to vaccination, and safety concerns. Results suggest our respondents harbored a high degree of skepticism regarding the efficacy and safety of the H1N1 vaccine, with less than 20% of respondents agreeing that “if you get an H1N1 vaccine you definitely will not get H1N1 influenza,” and a majority (64.4%) agreeing that “there are probably negative side-effects to getting the H1N1 vaccine.” Almost half (44.8%) of our respondents who chose to write a comment cited a safety concern. These findings are consistent with previous studies reporting concerns of side effects and perceived ineffectiveness of the vaccine as reasons for intent to become vaccinated against H1N1 [11, 12, 14, 32]. In sum, our results suggest that during the H1N1 pandemic, many college students considered receiving an H1N1 vaccine to be a risk or inconvenience that they were unwilling to accept. 

### 4.1. Limitations

The study is limited by use of a convenience sample that was heavily skewed toward white females. Studies of more representative samples of college students are necessary before findings can be generalized. This is especially important given findings of racial and ethnic differences in health beliefs regarding influenza vaccine decisions [33, 34]. Regression analyses are limited by the number of observations in the received vaccination group. Because the survey was conducted five months after the vaccine became available, there is a possibility of recall bias regarding vaccine decisions and influences on those decisions. Further, since this is a retrospective and correlational study, it is possible that health beliefs reported by the vaccinated group were influenced by the process of being vaccinated, rather than reflecting a prior, causal factor. 

### 4.2. Conclusions

Despite these limitations, the study has valuable implications for understanding college student responses to the recent influenza pandemic and for designing future vaccine programs targeting this population. If the next pandemic and vaccine are perceived as they were during the 2009-2010 H1N1 pandemic, low uptake among college students is likely to be repeated. Our findings suggest that messages based on increasing susceptibility may be less effective than those emphasizing that vaccination is an effective and safe means of preventing pandemic influenza infection, and that the H1N1 vaccine cannot cause disease or other harm. Further, obstacles to H1N1 immunization such as cost and convenience must be identified and addressed. 

The 2009 H1N1 pandemic occurred during an era of growing public attention to vaccine safety [35]. Because college students are a group at high risk of acquiring and spreading infectious diseases [36], understanding their immunization attitudes and behaviors is an important objective. The current findings suggest that many college students may have avoided H1N1 immunization during the 2009-2010 pandemic because they considered receiving the vaccine to lack clear benefits and to involve an unacceptable degree of risk. 

## Figures and Tables

**Table 1:** Predictors of having received an H1N1 vaccine: final logistic regression model^a^.

Variable	Adjusted logistic regression	
	Odds ratio	95% Confidence interval
Prior seasonal influenza vaccine acceptance	1.77^ ∗ ∗^	1.32–2.38
Perceived H1N1 disease susceptibility	1.13	
Perceived H1N1 disease severity	1.24	
Perceived H1N1 vaccine efficacy	2.40*	1.29–4.45
Perceived obstacles to obtaining H1N1 vaccine	.36*	.197–.66
H1N1 vaccine safety concerns	.47*	.29–.77
H1N1 vaccine endorsement of family and friends	1.01	

^a^Model contains all variables with significance of* P* < .10 in univariate logistic regression.

*P < .05; **P < .01.

**Table 2:** Qualitative results: influences on H1N1 vaccine decisions.

Category	Number of responses decisions	Influence on H1N1 vaccine	Representative example(s)^1^
Safety concern	39	Perceived dangers (direct or indirect) associated with H1N1 vaccination.	There are countless studies on vaccines being linked to autism in children and other studies done that have linked vaccinations to Alzheimer's. I believe that vaccinating everyone will provide a selective pressure for the virus to evolve further. I have seen a lot of cases with my friends where they get the vaccine and end up getting the flu because they are putting a “nonharmful” live virus into your body.

Severity	17	Beliefs regarding the severity of H1N1 influenza.	For something that is basically an overblown oversensationalized version of the common flu no thank you but I do not need a vaccine. If I get it, I get it. If I do not I do not. I am not really worried about it at all. Also if I get it, it will be a good workout for my immune system and I will just let nature run its course.

Medical dislike	16	General feelings toward medical procedures or environments.	My sisters and I have always refused taking cough syrup when my family gave it to us. I think that had a negative effect about me taking the vaccine. I hate hate hate (sic) getting shots.

Efficacy	15	Beliefs regarding effectiveness of the H1N1 vaccine in protecting against H1N1 influenza.	I believe that most of the H1N1 virus had already passed through and effected most people by the time the vaccine arrived. I most likely would not get this until I knew for sure that the results 100% provable!

Susceptibility	14	Perceptions of how widespread H1N1 is or one's personal likelihood of contracting H1N1.	I feel my immune system is strong enough that right now at my age I do not need a flu or H1N1 vaccine. I do not want the vaccine simply because the chances of me getting the H1N1 flu are not all that high as long as I take care of myself and sanitize.

Information	11	Adequacy of knowledge and information regarding H1N1 and the H1N1 vaccine.	I have not learned enough about the H1N1 vaccine to decide to get it for myself. My knowledge is limited—as I assume is the case for many students. Knowledge will influence whether or not a person is to get vaccinated.

Obstacles	9	Conditions that facilitate or interfere with obtaining an H1N1 vaccine.	I would only use free preventions. If it was a nasal spray that was given in our dorms I would get the vaccine.

History	6	Respondent's history of receiving seasonal flu or other vaccines.	I have never had a flu vaccine which is why I did not have the H1N1 vaccine. I nor any members in my family have ever gotten the seasonal flu shot.

Endorsement	5	Advice regarding H1N1 and vaccination.	My father who is a doctor has told me a few times that it really is not that big of a deal.

^1^ Spelling and punctuation errors present in respondent quotes have been corrected throughout this paper where they do not interfere with interpretation. No wording has been modified.
